# An Uncommon Presentation of Cervical Myelopathy

**DOI:** 10.7759/cureus.45609

**Published:** 2023-09-20

**Authors:** Alyssa N Nguyen, Ashley V Niu, Lauren F Ong, Neuzil Lai

**Affiliations:** 1 Neurology, California Northstate University College of Medicine, Elk Grove, USA; 2 Ophthalmology, California Northstate University College of Medicine, Elk Grove, USA; 3 Neurology, Kaiser Permanente South Sacramento Medical Center, Sacramento, USA

**Keywords:** degenerative myelopathy, compressive myelopathy, cervical flexion myelopathy, degenerative cervical myelopathy, spondylotic myelopathy, high cervical myelopathy, myelopathy, progressive myelopathy

## Abstract

Cervical myelopathy is a compressive spinal cord disease usually affecting individuals 55 and older. Involvement of C5-C7 is typical and classically presents with hand clumsiness, wide-based gait, and paresis. We present the case of a 38-year-old man with a pertinent history of a previous motor vehicle accident who presented to the emergency department for progressive numbness, weakness, and severe spasms in both lower extremities, and eventually developed bowel and bladder incontinence. Lumbar magnetic resonance imaging (MRI) showed moderate L3-L4/L5-S1 degenerative spinal changes; however, cervical MRI demonstrated severe C6-C7 spinal stenosis. The patient did not present with any upper extremity neurological changes. Given the relatively mild changes in the lumbar spine, the patient was concluded to have lower extremity and autonomic neurological issues due to severe cervical spinal stenosis. In this report, we present a relatively common case of cervical myelopathy and myelomalacia in a patient unusually presenting with no upper extremity signs and only lower extremity signs of progressive bilateral leg weakness and neurogenic urinary incontinence. This case emphasizes the importance of considering cervical spine workup in addition to thoracic and lumbar spine and conducting a comprehensive clinical neurological examination in the setting of lower extremity symptoms with progressive bilateral leg weakness and urinary incontinence.

## Introduction

Cervical myelopathy (CM) is a condition describing a compression of the spinal cord such as by spondylosis, ossification of the posterior longitudinal ligament, or disc herniation at the cervical level of the spinal column. Among adult spinal cord injuries (SCIs), 55% are at the cervical level (C1 to C7-T1), 15% at the thoracic level (T1-T11), 15% at the thoracolumbar level (T11-T12 to L1-L2), and 15% at the lumbosacral level (L2-S5). Furthermore, 40-50% of adult SCIs are caused by traffic accidents, 10-25% are work-related, 10-25% are caused by sports and recreation, 20% are falls-related, and 10-25% are violence-related [1]. There is no pathognomonic presentation of CM; thus, despite the prevalence of CM respective to all SCIs, the onset of CM can easily be overlooked or confounded as diagnosis heavily relies on elaborate and long-term clinical assessments of disease progression [2]. Symptoms of CM are correlated with the pathophysiology of upper motor neuron and lower motor neuron lesions, including upper or lower limb spasticity, hyperreflexia, pathological reflexes, and/or gait disturbance. CM most commonly manifests at C5-C6 or C6-C7, with the degeneration resulting in symptoms of hand numbness and hand clumsiness in the upper extremities [3]. Thoracic or lumbar radiculopathies are typically suspected in patients with neurological signs and symptoms in the lower extremities [4]. Thus, given the anatomy of a natural spinal cord and column, CM presenting with lower extremity symptoms (distal) and no upper extremity symptoms (proximal) can be an uncommon presentation in severe and progressive cases. Urinary incontinence is also a very late and uncommon presentation of compressive myelopathy.

We add to previous literature by presenting a rare case of CM and myelomalacia greatest at C6-C7 in a patient presenting without upper extremity deficits and only lower extremity signs, including progressive bilateral leg weakness and urinary incontinence.

## Case presentation

A 38-year-old man with a history of homelessness, polysubstance abuse disorder, attention-deficit/hyperactivity disorder, and post-traumatic stress disorder presented to the emergency department (ED) with symptoms of progressive numbness and weakness in both lower extremities, with the left being greater than the right. He reported numbness and weakness bilaterally in his lower extremities with severe spasms that progressively worsened over the last three months. The patient also suddenly developed severe neurogenic bowel, bladder hesitancy, and bladder incontinence. He had pre-existing, chronic hip pain due to a motor vehicle collision (MVC) six years before this visit. Despite records and imaging of the initial MVC visit being unavailable, the patient’s symptoms were unremarkable and stable leading up to this recent ED visit.

**Figure 1:**
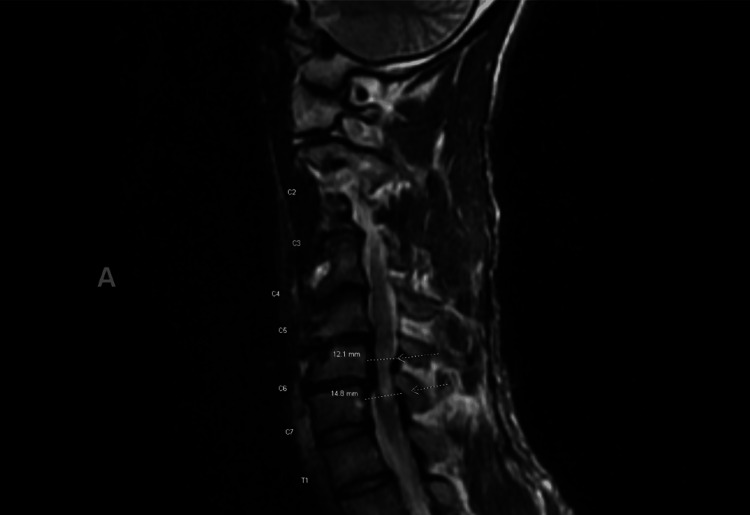
Cervical spine showing myelopathy and myelomalacia from severe spinal canal stenosis greatest at C6-C7 with chronic cord flattening from retrolisthesis and endplate osteophytes and ligamentum flavum buckling. T2-weighted sagittal view of MRI. Arrows indicate the extent of severity of canal stenosis with flattening of the cord.

Four months after the ED visit, the patient was seen by a neurology specialist. At this time, his impaired gait rapidly progressed from cane to wheelchair usage. Sudden spasm jerks in the legs were visible during the examination. Upon a full neurological physical examination, cranial nerves, strength, deep tendon reflexes, and sensation in the upper extremities were all relatively normal. Lower extremity examination findings were significant for slightly increased muscle tone bilaterally. On strength testing, lower extremities were 0/5 on direct testing, but 3/5 strength on pain withdrawal. Sensation was diminished at L2 and absent from L3 down. However, the patient grimaced at noxious stimuli to the inner thighs bilaterally. Deep tendon reflexes 2+ in lower extremities with plantar flexors were noted. The anal tone was decreased. Gait was deferred as the patient was wheelchair-bound. MRI of the cervical spine showed myelopathy and myelomalacia from severe spinal canal stenosis greatest at C6-C7 with chronic cord flattening from retrolisthesis and endplate osteophytes and ligamentum flavum buckling (Figure 1).

**Figure 2:**
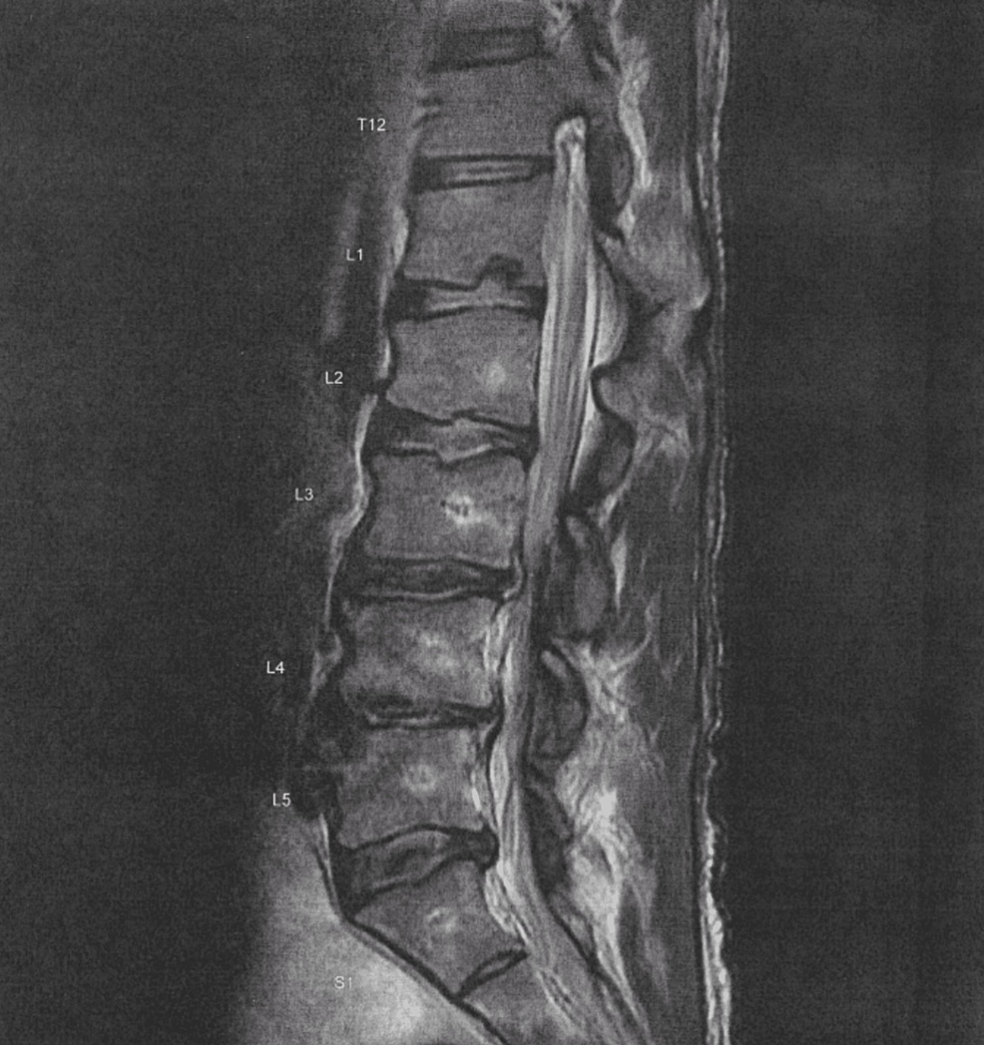
Lumbar spine showing moderate spondylotic and degenerative changes, most prominently L4-L5, less prominent L3-L4 and L5-S1, stable degenerative central spinal canal narrowing, stable small left paracentral disc herniation protrusion L2-L3, stable mild-to-moderate bilateral paracentral/neuroforaminal disc herniation protrusion and annular fissure L4-L5, stable small bilateral neuroforaminal and additional left paracentral disc herniation protrusion L3-L4, stable right paracentral and neuroforaminal disc herniation protrusion L5-S1.

For months leading up to the neurology consult, the patient had received a repeat MRI. MRI of the lumbar spine showed stable multilevel degenerative changes that were not convincing of infection, epidural collection, or significant spondylotic or degenerative changes contributing to the progression of signs or symptoms (Figure 2). Furthermore, the lumbar spine showed no fracture or suspicious bone marrow lesion; conus and cauda equina were normal.

Some differential diagnoses considered included vitamin B12 deficiency (subacute combined degeneration), Guillain-Barre syndrome (GBS), chronic inflammatory demyelinating polyradiculoneuropathy (CIDP), and hereditary spastic paraparesis. Due to his housing status, acquired subacute combined degeneration complicated by vitamin B12 deficiency was initially considered as the etiology for his paraplegia and spasticity. However, vitamin levels were in the lower range of normal, making B12 deficiency less likely. GBS was ruled out due to the timeline of his presenting symptoms, which would be expected to be seen more acutely. Further, the lack of hyporeflexia lowered GBS and CIDP on the differential. Hereditary spastic paraparesis was unlikely as numbness would be uncommon. Furthermore, it is commonly associated with hyperreflexia and has a genetic component which the patient denied having any known family history of. The patient was unable to tolerate neurophysiology evaluations or lumbar punctures; however, laboratory workup, brain CTs, spine MRIs, and lack of sensory changes deemed the aforementioned pathologies unlikely.

Upon extensive hospitalization and neurology/neurosurgery consultation, the patient was recommended to undergo non-urgent cervical spine surgery. However, after the consultation, the patient was lost to follow-up.

## Discussion

Several studies have examined the prevalence, or lack thereof, of myelopathic signs and symptoms to localize cervical spinal cord lesions. Niu et al. found that in a large surgical cohort of patients with CM, the most common overall symptoms experienced were both upper and lower extremity motor deficits (82.6% and 81.2%, respectively) with a chief complaint of upper extremity sensory deficit (46.5%) [5]. In a prospective case-control study, Rhee et al. warned that although myelopathic signs are common in CM patients, they may be negative in approximately one-fifth, and further imaging and/or tests should be obtained to confirm a diagnosis [6]. In exploring these “atypical” CM patient presentations, Funaba et al. found that a small proportion of patients with C6-C7 myelopathy had no to few neurological signs in the upper limbs, and only presented with neurologic symptoms of gait disturbance in their lower limbs. This 2015 study noted that clinical and electrophysiological findings associated with cervical myelopathy relative to expected spinal cord anatomy still remain unclear and that there are few studies to date that delve into this anomaly [7].

This patient was seen in the ED for sensory changes in both lower extremities, which were reasonably attributed to previously established lumbar spondylotic and degenerative changes and warranted obtaining lumbar and sacral spine MRI. However, the mild to insignificant lumbar changes seen on MRI did not clinically correlate to the severity of lower limb and autonomic sensory changes. Four months later, a full neurology consult with cervical spine MRI revealed severe spinal canal stenosis greatest at C6-C7, thereby suggesting an uncommon case of CM with lower extremity manifestations in the absence of upper extremity signs. While lumbar herniation at L3-4/L5-S1 was noted, its potential involvement in the progression of symptoms cannot be ruled out. Relevant pathologies that would present similarly to this patient include lumbar lesions such as cauda equina syndrome or radiculopathy, or even more rarely, conus medullaris syndrome. Due to the protective anatomy of the lumbar spine, fractures at the levels of conus medullaris and cauda equina often present with incomplete neurological deficits [8]. Despite lumbar spine imaging for this patient being relatively unremarkable with normal conus and cauda equina and no fracture, further neurological investigation was warranted. The patient presented with urinary incontinence in the setting of a spinal cause, which should also be considered as a possible cause of urinary incontinence in the initial visit. The cervical spinal cord contains nerves responsible for transmitting signals including lower extremities, so compression of C6-C7 could have affected motor and sensory function in lower extremities. Kok et al. highlighted two cases of T11-T12 cord lesions that presented as pure lower motor neuron syndromes. Similarly, C6-C7 cord lesions could affect lower extremity spinal nerves from vascular insufficiency in anterior horn cells leading to the progression of lower extremity signs and symptoms initially [9]. In addition, there possibly could have been upper motor neuron signs that would have pointed toward a cord lesion above the conus; thus, it is important to perform a detailed and complete comprehensive neurological examination. Although conducting a neurological examination may be considered challenging to most physicians, it remains a crucial part of patient care. A comprehensive evaluation is essential because neurological conditions can manifest in various ways, making it an invaluable tool for determining differential diagnoses [10]. Especially given the circumstance of disjointed patient care and unreliable adherence due to complex social and medical history, thorough workups and imaging studies for lower extremity spinal cord symptoms should be explored in not only the thoracic and lumbar spine but also in the cervical spine [11].

## Conclusions

In this report, we present an uncommon and complex case of CM and myelomalacia greatest at C6-C7 in a patient presenting with only lower extremity signs of progressive bilateral leg weakness, urinary incontinence, and no upper extremity signs. This case emphasizes the importance of conducting a comprehensive clinical neurological examination and cervical spine evaluation, along with thoracic and lumbar spine assessments, when presented with lower extremity symptoms accompanied by progressive bilateral leg weakness and urinary incontinence. Notably, even in the absence of signs and symptoms in the upper extremities, a high index of suspicion of a cervical cord pathology and a thorough investigation of the entire spine is crucial.
